# Assessment of Therapeutic Responses Using a Deep Neural Network Based on ^18^F-FDG PET and Blood Inflammatory Markers in Pyogenic Vertebral Osteomyelitis

**DOI:** 10.3390/medicina58111693

**Published:** 2022-11-21

**Authors:** Hyunkwang Shin, Eunjung Kong, Dongwoo Yu, Gyu Sang Choi, Ikchan Jeon

**Affiliations:** 1 Department of Information and Communication Engineering, Yeungnam University, Gyeongsan 38541, Republic of Korea; 2 Department of Nuclear Medicine, Yeungnam University College of Medicine, Daegu 42415, Republic of Korea; 3 Department of Neurosurgery, Yeungnam University College of Medicine, Daegu 42415, Republic of Korea

**Keywords:** pyogenic, vertebral osteomyelitis, therapeutic response, FDG-PET, deep neural network

## Abstract

*Background and Objectives:* This study investigated the usefulness of deep neural network (DNN) models based on ^18^F-fluorodeoxyglucose positron emission tomography (FDG-PET) and blood inflammatory markers to assess the therapeutic response in pyogenic vertebral osteomyelitis (PVO). *Materials and Methods:* This was a retrospective study with prospectively collected data. Seventy-four patients diagnosed with PVO underwent clinical assessment for therapeutic responses based on clinical features during antibiotic therapy. The decisions of the clinical assessment were confirmed as ‘Cured’ or ‘Non-cured’. FDG-PETs were conducted concomitantly regardless of the decision at each clinical assessment. We developed DNN models depending on the use of attributes, including C-reactive protein (CRP), erythrocyte sedimentation ratio (ESR), and maximum standardized FDG uptake values of PVO lesions (SUV_max_), and we compared their performances to predict PVO remission. *Results:* The 126 decisions (80 ‘Cured’ and 46 ‘Non-cured’ patients) were randomly assigned with training and test sets (7:3). We trained DNN models using a training set and evaluated their performances for a test set. DNN model 1 had an accuracy of 76.3% and an area under the receiver operating characteristic curve (AUC) of 0.768 [95% confidence interval, 0.625–0.910] using CRP and ESR, and these values were 79% and 0.804 [0.674–0.933] for DNN model 2 using ESR and SUV_max_, 86.8% and 0.851 [0.726–0.976] for DNN model 3 using CRP and SUV_max_, and 89.5% and 0.902 [0.804–0.999] for DNN model 4 using ESR, CRP, and SUV_max_, respectively. *Conclusions:* The DNN models using SUV_max_ showed better performances when predicting the remission of PVO compared to CRP and ESR. The best performance was obtained in the DNN model using all attributes, including CRP, ESR, and SUV_max_, which may be helpful for predicting the accurate remission of PVO.

## 1. Introduction

Pyogenic vertebral osteomyelitis (PVO) invades the spine and adjacent structures, shows non-specific symptoms, and is usually progresses to destructive spondylodiscitis with abscess formation at the time of diagnosis [1,2,3,4]. Long-term intravenous antibiotics are generally recommended for 6 to 12 weeks for treating PVO, but treatment guidelines have not been clearly established due to the diversity of causative bacteria and antibiotic resistance in the regions [2,5,6,7,8]. Moreover, in the assessment of therapeutic response, blood inflammatory markers such as C-reactive protein (CRP) and erythrocyte sedimentation rate (ESR) can be easily influenced by other physical conditions. Magnetic resonance imaging (MRI), regarded as the best modality to present the anatomical state of the spine, also has limitations in differentiating residual PVO and post-treatment structural abnormalities under the healing process [4,9,10].

To overcome these limitations, there has been new attempt to apply ^18^F-fluorodeoxyglucose positron emission tomography (FDG-PET) in the assessment of the therapeutic response in PVO [11,12,13]. Changes in FDG uptake, presented as maximum standardized FDG uptake (SUV_max_), showed superior outcomes for evaluating residual PVO compared to CRP and ESR [12,13,14]. However, SUVmax can also be variable depending on the structural features, including the anatomical location of major PVO lesions and the stabilization of the damaged intervertebral discs (autofusion) in cured PVO after sufficient antibiotic therapy [15,16]. These characteristics can also cause variability in the SUVmax, even in patients with cured PVO, and, therefore, blood inflammatory markers should also be considered for the assessment of therapeutic responses.

Deep learning (DL) is an emerging technique, and its application is gradually increasing in the field of medicine. DL differs from traditional machine learning methods, such as linear regression, artificial neural network, support vector machines, and naïve Bayes classifiers, by how representations are automatically discovered from raw data. The algorithms of DL use multiple deep layers of perceptron that capture low- and high-level representations of data, enabling them to learn richer abstractions of inputs [17,18]. In particular, a deep neural network (DNN), a type of DL, is a mathematical model that simulates the structure and functionalities of a biological neural network. It is a feed-forward neural network with multiple hidden layers, which can be applied in the problems of classification and regression [19]. In particular, a DNN is a relatively simple algorithm compared to other DL techniques and shows excellent performance for clinical predictions by binary classification based on various clinical features.

In this study, we used DNN to overcome difficulties in obtaining high objectivity for the assessment of therapeutic responses in PVO. DNN models were developed based on various combinations of SUV_max_, ESR, and CRP, considering their relationships and complementary effects, and their performances were evaluated and compared to predict the remission of PVO after antibiotic therapy.

## 2. Patients and Methods

### 2.1. Patients

We retrospectively reviewed the prospective collected clinical and radiological data of 100 patients with PVO (63 men and 37 women) treated at single tertiary university hospital from December 2017 to March 2021. The criteria of inclusion were as follows: (1) patients presented with clinical symptoms (fever, back pain, or neurological signs) and specific findings on MRI of PVO, (2) PVO was on thoracolumbar spine, (3) with/without identification of causative bacteria in the PVO lesion or ≥2 sets of blood cultures, and (4) above 20 years old. Patients with tuberculous vertebral osteomyelitis, a PVO lesion containing instrumentation or bone cement, bone infection other than in the spine, recent trauma, tumor, pregnancy, or experiencing severe concomitant medical problems were excluded.

Under the voluntary written informed consent, all patients participated in this study to receive a simultaneous FDG-PET/MRI at each clinical assessment during antibiotic therapy. All clinical and radiological data were collected and analyzed under the approval of the institutional review board.

### 2.2. Clinical Assessment and Determining Therapeutic Response

All patients participating in this study underwent clinical assessments to determine therapeutic responses during antibiotic therapy based on clinical symptoms including fever, back pain, and CRP (normal range <0.5 mg/L in our institute), which were performed after the minimum intravenous antibiotic therapy of 3 weeks [20]. In addition, each clinical assessment was conducted in the absence of any other medical problems that could affect the decision making. The decisions for therapeutic responses by clinical assessments were classified as ‘Cured (group C)’ or ‘Non-cured (group NC)’. Simultaneous FDG-PET/MRIs of the spine involving PVO were taken concurrently at each clinical assessment, regardless of the decision for therapeutic response. The choice of selective or empirical parenteral antibiotics was made after consultation with an infectious disease physician.

At the clinical assessment, decisions based on the condition presenting with sustained or aggravated clinical symptoms, sustained or re-elevated CRP, and aggravation of the PVO lesion on MRI (defined as newly developed or progressed epidural/intravertebral/psoas abscess) and/or persistent causative bacteria on the PVO lesion were classified into group NC. When the decision was classified into group C by clinical assessment, antibiotic therapy was discontinued. The decision of group C was observed with a minimum follow-up period of six months after discontinuing antibiotic therapy, and they were finally confirmed as group C if there was no recurrence [21]. Recurrence was defined as the condition presenting with a re-elevation of CRP ≥1 mg/L, aggravated clinical symptoms with/without fever, and aggravation of the PVO lesion on MRI during the follow-up period. If there was a recurrence in the initial decision of group C during the follow-up period, the follow-up was stopped and the decision was finally classified into group NC.

### 2.3. FDG-PET/MRI and Image Analysis

Each patient underwent simultaneous FDG-PETs/MRIs (Biograph mMR; Siemens Healthcare, Erlangen, Germany) with fasting for more than 6 h to maintain a blood glucose level of under 8.9 mmol/L before the intravenous administration of FDG (3.7 MBq/kg). The acquisition of simultaneous FDG-PETs/MRIs was initiated 60 min after FDG injection, and the thoracolumbar spine centering PVO lesion was scanned under one–two bed positions with the approved surface coil. FDG-PET data acquisition was performed over 20 min, and the MRI data were also simultaneously obtained based on the predetermined sequence protocol [14]. We applied a 3-dimensional ordered subsets expectation maximization iterative reconstruction (OSEM-IR) algorithm with 3 iterations and 21 subsets for the FDG-PET data using a 172 × 172 matrix. To measure the FDG uptake value, we drew an ellipsoid volume of interest including the PVO lesion based on the spine structures of the MRI and confirmed the maximum standardized uptake value of FDG (SUV_max_).

### 2.4. Deep Neural Network Model

We developed DNN models as pattern classifiers to predict the remission of PVO using supervised learning. This DNN is a type of feed-forward artificial neural network whereby logical units of one layer only communicate with the subsequent layer, and it consists of three kinds of layers including input, hidden, and output layers. DNN models were developed based on various combinations of the attributes including ESR, CRP, and SUV_max_. The DNN model utilized the backpropagation rule for training, which repetitively calculates the error function for each input and backpropagates the error to the previous layer. The weights were adjusted in direct proportion to the error in the neural nodes to which it was connected. The data of the ESR, CRP, and SUV_max_ were randomly assigned as 70% to the training set and 30% to the test set, respectively. The DNN model was developed using Keras 2.6.0 with a TensorFlow 2.6.0 backend, and all experiments were performed based on a single Nvidia GeForce RTX 2080 Ti graphics card.

### 2.5. Statistical Analysis

We used SPSS version 25.0 software (SPSS Inc., Chicago, IL, USA) for conducting the statistical analyses. Categorical and continuous variables are presented as numbers with percentages and median values with a range, respectively. AUCs were used to assess the performance of the DNN models to predict the remission of PVO. To compare the two population means, the Kolmogorov–Smirnov test was used to determine whether the sample data had a normal distribution (normality test), and then the Student’s *t*-test and Mann–Whitney U test were used for parametric and non-parametric continuous variables, respectively. Probability values (*p*-values) of less than 0.05 were considered statistically significant.

## 3. Results

### 3.1. Demographic and Clinical Data

Among 100 patients, 26 were finally excluded from this study due to the following reasons: only a back muscle abscess with no spondylodiscitis (*n* = 8), spinal screw within the PVO lesion (*n* = 6), tuberculous spondylodiscitis (*n* = 3), degenerative change (*n* = 1), trauma (*n* = 2), ankylosing spondylitis (*n* = 2), other severe concomitant medical problems (*n* = 1), and lost to follow-up or withdrawal of participation (*n* = 3). Final analyses were performed on 74 patients (47 men and 27 women) with a mean age of 67.27 ± 11.18 (37–85) years. The main cause of PVO was injection or acupuncture (47.3%, 35/74). Back pain was the most common symptom (97.3%, 72/74) in the initial clinical features of PVO, and fever was present in half of the patients with PVO (50.0%, 37/74). The lumbar-sacral spine was the main location of the PVO (87.8%, 65/74). Detailed data are presented in Table 1.

### 3.2. Causative Bacteria and Antibiotic Therapy

The rate of bacterial identification was 51.4% (38/74) in the blood and/or PVO tissue cultures. The main causative bacterium identified was methicillin-sensitive *Staphylococcus aureus* (34.2%, 13/38). The mean duration of parenteral antibiotic therapy was 44.14 ± 16.70 (21–89) days. Detailed data are presented in Table 2.

### 3.3. Clinical Assessment and Determination of Therapeutic Response

**Figure 1:**
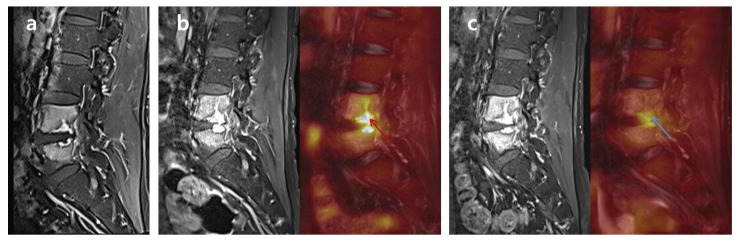
Differences in FDG uptake between ‘Non-cured’ and ‘Cured’ FDG-PET/MRIs. A 54-year-old male patient with lumbar PVO on L4-5 was treated with vancomycin and ciprobay for 46 days. The first clinical assessment on the 25th day of antibiotic therapy presented sustained back pain, intermittent fever, and a CRP/ESR of 1.399/64 (‘Non-cured’). Compared to the initial MRI (**a**) performed at the other hospital, the first FDG-PET/MRI (**b**) revealed an elevated FDG uptake (red arrow; SUV_max_ 6.58) and progression of the PVO lesion. After additional antibiotic therapy, the patient showed improved back pain and no fever, with a CRP/ESR of 0.325/31 in the second clinical assessment on the 46th day of antibiotic therapy (‘Cured’). The second FDG-PET/MRI (**c**) also shows markedly decreased FDG uptake and a reduced territory of the PVO lesion (blue arrow; SUV_max_ 4.3). However, determination of the therapeutic response seemed to be impossible based on the MRI due to the continuous enhancement of the PVO lesion even after successful treatment. FDG ^18^F, fluorodeoxyglucose; FDG-PET/MRI ^18^F, fluorodeoxyglucose positron emission tomography/magnetic resonance imaging; PVO, pyogenic vertebral osteomyelitis; CRP, C-reactive protein (mg/dL); ESR, erythrocyte sedimentation rate (mm/h); SUV_max_, maximum standardized uptake value of FDG.

**Figure 2:**
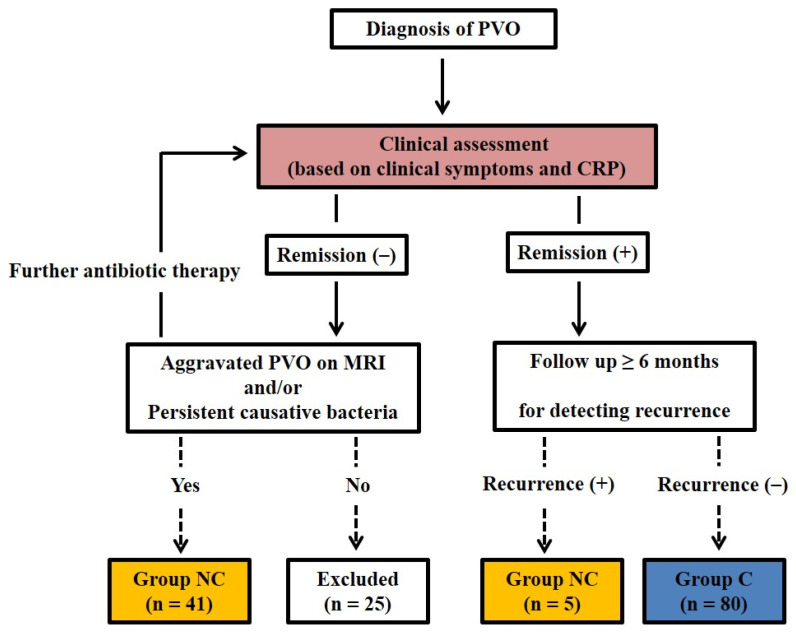
Flowchart of the decision classification in the clinical assessment. Among the decisions with residual PVO (no remission), the decisions showing the presence of causative bacteria and/or an aggravated PVO lesion on MRI were only classified into group NC (*n* = 41), and the others (*n* = 25) were excluded from the study. The 85 decisions with remission were followed-up with a minimum of six months after discontinuing antibiotic therapy, and the 80 decisions with no recurrence were finally classified into group C. The five recurrences were classified into group NC. Therefore, the final analysis was performed with 80 patients in group C and 46 in group NC. PVO, pyogenic vertebral osteomyelitis; MRI, magnetic resonance imaging; Group C, cured; Group NC, non-cured.

Among the decisions of residual PVO (no remission), those showing a definite aggravation of the PVO lesion on MRI and/or persistent causative bacteria on the PVO lesion were classified into group NC (*n* = 41), and the others (*n* = 25) with no aggravation of the PVO lesion on MRI and/or persistent causative bacteria on the PVO lesion were excluded due to the possibility of false positives for residual PVO caused by other general conditions or the subjectivity of clinical symptoms. The 84 decisions with remission were followed-up for 12.66 ± 8.81 (1–44) months. The 80 decisions with no recurrence were finally classified into group C, and 5 decisions with recurrence were classified into group NC. These five recurrences occurred at 2.20 ± 1.44 (1–4.5) months during the follow-up period. Therefore, the final analysis was performed with 80 patients in group C and 46 patients in group NC (Figure 1 and Figure 2). There were statistically significant differences in the ESR, CRP, and SUV_max_ between groups C and NC (*p* < 0.001) (Table 3).

### 3.4. Development of DNN Model to Predict Remission of PVO

**Figure 3:**
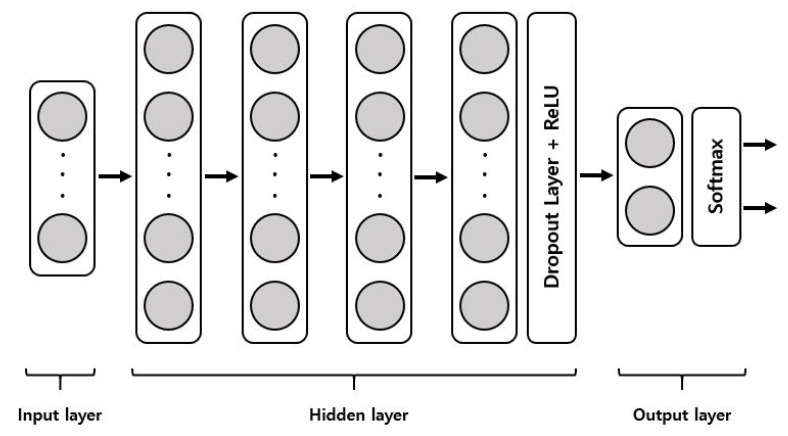
Structure of our DNN model.

The 126 decisions based on the clinical assessments were randomly divided into 70% for the training set (88 decisions; 56 in group C and 32 in group NC) and 30% for the test set (38 decisions; 24 in group C and 12 in group NC). The ESR, CRP, and SUV_max_ were used as the attributes. We developed four DNN models, depending on the involved attributes to predict remission of PVO, as follows: DNN model 1 with ESR and CRP, DNN model 2 with ESR and SUV_max_, DNN model 3 with CRP and SUV_max_, and DNN model 4 with ESR, CRP, and SUV_max_. The performances of four DNN models were compared under the same conditions. A rectifier linear unit (ReLu) for the activation functions in four hidden layers, softmax cross-entropy to calculate the loss, and adaptive moment estimation for loss optimization with a learning rate of 0.001 were adopted. The dropout technique was used in the output layer to prevent overfitting with the training set. Our DNN models are summarized in Figure 3.

Here, we summarize the structure of the DNN developed in this study for the detection of residual PVO. The DNN consists of an input layer, multiple hidden layers, and an output layer. The input layer feeds clinical data consisting of input features x∈{x_1_, …, x_n_} to the first hidden layer, where *n* is the number of input features. The hidden layer consists of four layers; the first layer has 32 neurons, the second layer has 16 neurons, the third layer has 8 neurons, and the fourth is a ReLU activation layer. The last hidden layer contains five neurons, followed by a dropout for regularization and a ReLU for activation. The output layer generates a probability distribution of the predictions using softmax cross-entropy activation. Adam for loss optimization with a learning rate of 0.001 was adopted (DNN, deep neural network; PVO, pyogenic vertebral osteomyelitis; ReLU, rectifier linear unit; and Adam, adaptive moment estimation).

### 3.5. Performances of the DNN Models for Predicting the Remission of PVO

**Figure 4:**
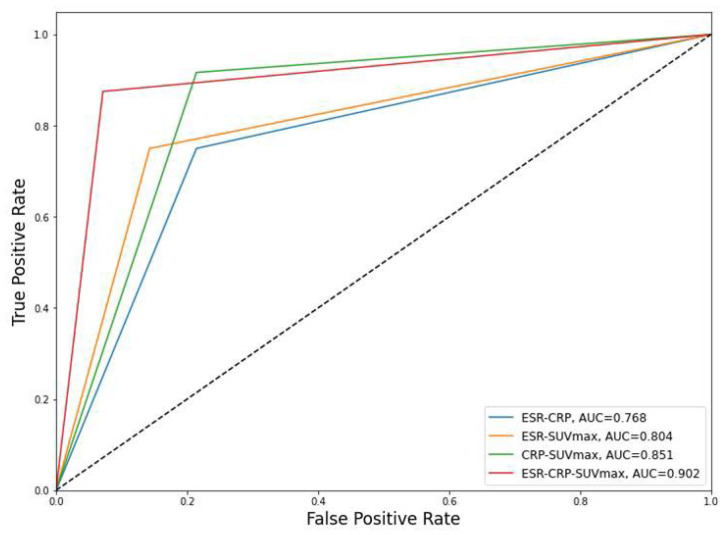
AUCs of the DNN models. CRP, C-reactive protein (mg/dL); ESR, erythrocyte sedimentation rate (mm/h); SUV_max_, maximum standardized uptake value of FDG; AUC, area under the receiver operating characteristic curve.

The performances of each DNN model were compared when they were trained at 100 epochs. The sensitivity, specificity, positive predictive value (PPV), negative predictive value (NPV), accuracy, and AUCs were 75%, 78.6%, 85.7%, 64.7%, 76.3%, and 0.768 [95% confidence interval, 0.625–0.910] in DNN model 1; 75%, 85.7%, 90%, 66.7%, 79%, and 0.804 [0.674–0.933] in DNN model 2; 91.7%, 78.6%, 88%, 84.6%, 86.8%, and 0.851 [0.726–0.976] in DNN model 3; and 87.5%, 92.9%, 95.5%, 81.3%, 89.5%, and 0.902 [0.804–0.999] in DNN model 4. The performances of the DNN models are summarized in Table 4 and Figure 4.

### 3.6. Incorrectly Predicted Cases in the DNN Model 4

DNN model 4 using ESR, CRP, and SUVmax showed the best performance with approximately 90% accuracy to predict the remission of PVO. There were 4 incorrect predictions among the 38 cases in the test set. DNN model 4 showed opposite predictions for three cases in group C and one case in group NC. The detailed data are presented in Table 5.

## 4. Discussion

To date, the assessment of therapeutic responses in PVO has been performed mainly based on changes in clinical symptoms and blood inflammatory markers. However, no clear standards have yet been established. Generally, compared with ESR and white blood cell (WBC) count, CRP was highly correlated with clinical symptoms and rapidly decreased as the clinical condition improved [22]. In particular, WBC count is known to be less useful for applications related to diagnosis and the assessment of therapeutic responses because WBC count often provides false-negative results despite the presence of PVO in elderly or immunocompromised patients [23,24]. In addition, there are also no definite guidelines for antibiotic therapy due to frequent negative cultures for causative bacteria and various causative bacteria with different antibiotic resistance rates by region. Kim et al. [5] reported that identifying the causative bacteria was possible in only half of all patients. *S. aureus* was the main causative bacteria, and approximately 40% had methicillin resistance. For the treatment of PVO, an average of 6 weeks of parenteral antibiotics is usually recommended, although there some studies that considered 2–4 weeks sufficient [2,25,26,27]. Here, a clinical assessment based on clinical symptoms and a CRP was performed after more than 3 weeks of intravenous antibiotic therapy.

The application of FDG-PET to assess therapeutic responses in PVO has been attempted for the last 10 years [12,13,14]. The literature has reported that FDG-PET was affected less by other general conditions and, therefore, it objectively represents PVO lesions compared to blood inflammatory markers [12]. In particular, Jeon et al. [14] confirmed that FDG-PET, using the degree of FDG uptake, had higher accuracy compared to a CRP, an ESR, and an MRI for detecting residual PVO. The difference in FDG uptake mediated by glucose transporters in the cell membrane, depending on the phases of PVO, can be explained based on the pathophysiological features of osteomyelitis [28,29,30]. Activated neutrophils accumulate in the early phase, which shows high glucose consumption for chemotaxis and phagocytosis. Lymphocytes, plasma cells, histiocytes, and some polymorphonuclear leukocytes are predominant inflammatory cells in the chronic or recovery phase and have low glucose consumption. In addition, fibrosis and granulation tissues form around the foci of inflammation and bone marrow during the recovery phase, and there are fatty changes, increased new bone formation by osteoblasts, and dilated blood vessels. The mechanical stress on intervertebral structures including the intervertebral disc and endplates associated with the patient’s activities, in addition to the changes during the recovery phase, can result in a sustained increase in localized FDG uptake at the intervertebral structures, even after successful treatment, compared to widespread FDG uptake when residual PVO continues [14].

However, FDG uptake presented as SUV_max_ also showed some variability, although it was less than that observed for CRP. Here, we discuss the reasons for the abnormally increased FDG uptake on the FDG-PET imaging of cured PVO, which was obtained at the time of antibiotic therapy discontinuation and showed no recurrence during the follow-up [15,16]. First, when the main PVO lesions containing SUV_max_ were located in the bone marrow within the vertebral body or presented as a form of intramuscular abscess, the SUV_max_ was higher than that observed on the intervertebral structures. However, the value of SUV_max_ was not related to the clinical symptoms, and the destruction of the intervertebral structure as the main PVO lesion was the main cause of the sustained back pain, even after successful treatment. Second, when the damaged intervertebral structure of the PVO was healed by autofusion with a loss of joint function during the follow-up period, a higher SUV_max_ was observed in the intervertebral structure, including the endplate and disc on FDG-PET imaging, of those with cured PVO. We hypothesize that these various features of FDG uptake on the FDG-PET imaging of cured PVO patients might be an important reference for interpreting the value of SUV_max_ to assess therapeutic responses. However, considering the variability in FDG uptake as described above, FDG-PET is still limited for use as an absolute standard method to assess therapeutic responses in PVO. It will be effective to supplement FDG-PET with hematological inflammatory makers, which are still used as a popular treatment method. As a result, we analyzed the performance of ESR, CRP, and SUVmax applied together based on a DNN in this study.

Back pain is one of the main clinical symptoms of PVO and can be considered an indicator for assessing therapeutic responses in PVO. However, back pain can easily be influenced by individual subjective factors or psychological status. It is difficult to measure back pain objectively and the reliability of the measurement result may be low. In this study, we developed DNN models using only automatically and objectively measured ESR, CRP, and SUV_max_ to increase the reliability of the results. All DNN models developed in this study were started to overfit with the presenting estrangement of loss between the training and test sets at approximately 100 epochs, and there was a decrease in accuracy with training at over 100 epochs. Therefore, a comparison of the performances between the DNN models depending on the use of attributes was possible and appropriate at 100 epochs. The best performance was obtained with DNN model 4, which had an accuracy of 89.5% and an AUC of 0.902. DNN model 1 had the lowest performance, with an accuracy of 76.3% and an AUC of 0.768 when using ESR and CRP. Of note, ESR and CRP, currently used as important measurement methods at clinical assessment, were confirmed to have the lowest performance even after using deep learning. These results might be explained by the fundamental limitations of ESR and CRP themselves, which are susceptible to other general conditions. Overall, the DNN models that included SUV_max_ as an attribute showed better performances than models without it. It is thought that the FDG uptake of FDG-PET also indicated the status of PVO lesions more objectively than ESR and CRP under a predictive DNN model, as confirmed in previous studies using conventional statistics.

DNN model 4 based on the ESR, CRP, and SUVmax as attributes showed approximately 90% accuracy for predicting the remission of PVO, and there were incorrect predictions in 4 out of 38 cases in the test set. DNN model 4 showed opposite predictions for three cases in group C and one case in group NC. In two cases in group C (case numbers 33 and 107), there were sustained increases in ESR and CRP levels, with a moderate elevation of the SUV_max_. These are likely to be judged as ‘Non-cured’ using blood inflammatory markers in the currently applied clinical assessment. In one other case in group C (case number 45), a markedly elevated SUV_max_ value led to a ‘Non-cured’ decision under DNN model 4, although it was highly likely to be judged as ‘Cured’ based on the ESR and CRP levels by clinical assessment. An elevated SUV_max_ value was identified in three out of four incorrect predictions. Based on the aforementioned theory, we may consider the reasons for elevated SUV_max_ values in terms of the location of the main PVO lesion and autofusion. However, although these analyses of the characteristics of PVO lesions can help to understand elevated SUV_max_ values in cases with cured PVO, additional studies with more cases are required to apply these theories to the DNN model for predicting the remission of PVO. Considering the possibility of false-positives of SUV_max_ as seen for the above incorrect predictions, blood inflammatory markers can partially contribute to achieving improved performance in a DNN model by compensating for the shortcomings of the SUV_max_. The last incorrect prediction of group NC (case number 126) with normalized values of ESR and CRP and a markedly decreased SUV_max_ was suitable to be determined as ‘Cured’ in the clinical assessment and with DNN model 4, although residual PVO persisted. This was an unpredictable case and this shortcoming should be overcome to improve the performance of DNN models in future studies.

Our study had several limitations. First, decision making for therapeutic responses in this study was conducted based on the existing method currently used in the medical field. There is still no definite method to confirm the presence of residual PVO in group NC, although patients in group C can finally be determined as ‘Cured’ with a sufficient follow-up period. This problem remains a major limitation in many studies of PVO. In this study, we limited cases to group NC only when there was a definite aggravation of the PVO lesion on MRI and/or persistent causative bacteria on the PVO lesion under the additional bacterial culture. The decision of ‘Non-cured’, which did not meet these conditions (the possibility of false positives for residual PVO), was excluded to increase the reliability of the study. Second, this study was conducted based on a relatively small number of FDG-PET images compared with recent studies using deep learning. The application of FDG-PET for diagnosis and assessing therapeutic responses in PVO has not yet been generalized, and it is not easy to conduct a large-scale study considering the cost of FDG-PET. However, based on the high accuracy of using FDG-PET to assess therapeutic responses in PVO, as demonstrated in previous studies, we think that the AUC observed in this small-scale study will be reproduced in large-scale studies. Lastly, this study was conducted in a single center and our results were not confirmed under the same conditions as those in other institutions. Multicenter research is an important factor to obtain high reliability of research results. Unfortunately, this study was conducted with FDG-PET/MRI for various reasons, including the measurement of SUV_max_ based on the exact anatomical structure of the PVO lesion under MRI, and the availability of FDG-PET/MRI is still poor compared that of FDG-PET/CT. To overcome these limitations and clearly confirm the usefulness of FDG-PET in PVO, additional multi-center studies with a larger number of participants are required.

## 5. Conclusions

DNN models using SUV_max_ had a better performance for predicting the remission of PVO compared to those using CRP and ESR. However, the best performance was obtained in a DNN model using all attributes, including CRP, ESR, and SUV_max_, which may be achieved by compensating for the limitations of each attribute. We expect that the use of a DNN model based on a combination of FDG-PET and blood inflammatory markers may help accurately predict remission in PVO.

## Figures and Tables

**Table 1:** Demographic and clinical data.

Characteristics	Values
Age, years	67.27 ± 11.18 (37–85)
Sex (Male/Female)	47/27
Cause of PVO	
Spontaneous	30/74 (40.5%)
Procedure-related	44/74 (59.5%)
Injection or acupuncture	35/44 (79.5%)
Operation	9/44 (20.5%)
Initial clinical features at diagnosis of PVO	
Fever (°C, >37.3)	37/74 (50.0%)
Back pain	72/74 (97.3%)
Radiculopathy	39/74 (52.7%)
Weakness	10/74 (13.5%)
Bowel and bladder symptoms	4/74 (5.4%)
Extent of PVO, levels	1.35 ± 0.53 (1–3)
Location of PVO	
Thoracic spine	6/74 (8.1%)
Thoracic-lumbar spine	3/74 (4.0%)
Lumbar-sacral spine	65/74 (87.8%)
ESR (mm/h)	67.68 ± 30.21 (6–120)
CRP (mg/L)	9.84 ± 9.16 (0.03–33.8)
Duration of follow up, months ^#^	12.66 ± 8.81 (1–44)

PVO, pyogenic vertebral osteomyelitis; ESR, erythrocyte sedimentation ratio (normal range of <20 mm/h); CRP, C-reactive protein (normal range of <0.5 mg/dL), ^#^ after discontinuing of antibiotic therapy under the decision of ‘Cured’.

**Table 2:** Microorganisms and antibiotics.

Characteristics	Values
Identification of causative bacteria	38/74 (51.4%)
Causative bacteria	
Gram-positive bacteria	35/38 (92.1%)
*Staphylococcus aureus*	18/35 (51.4%)
MSSA	13/15 (60.0%)
MRSA	5/15 (40.0%)
*Coagulase-negative staphylococci*	6/35 (17.1%)
MRSE	3/6 (50.0%)
Others	3/6 (50.0%)
*Streptococcus species*	7/35 (20.0%)
*Enterococcus species*	4/35 (11.4%)
Gram-negative bacteria	3/38 (7.9%)
Escherichia coli	2/3 (66.7%)
*Enterobacter species*	1/3 (33.3%)
Non	36/74 (48.6%)
Routes of causative bacterial diagnosis	
Blood	10/38 (26.3%)
PVO lesion	19/38 (50.0%)
Blood and PVO lesion	6/38 (15.8%)
Duration of parenteral antibiotics, days	44.14 ± 16.70 (21–89)

MSSA, methicillin-sensitive *staphylococcus aureus*; MRSA, methicillin-resistant *staphylococcus aureus*; MRSE, methicillin-resistant *staphylococcus epidermidis*; PVO, pyogenic vertebral osteomyelitis.

**Table 3:** Clinical features between groups C and NC.

Attributes	Group C (*n* = 80)	Group NC (*n* = 46)	Total (*n* = 126)
ESR *	42.64 ± 27.76 (7–120)	71.57 ± 31.36 (7–120)	53.20 ± 32.20 (7–120)
CRP *	0.80 ± 1.07 (0.02–5.93)	3.01 ± 3.20 (0.11–15.75)	1.61 ± 2.36 (0.02–15.75)
SUV_max_ *	4.59 ± 2.15 (1.66–16.11)	7.30 ± 2.14 (3.61–14.65)	5.58 ± 2.51 (1.66–16.11)

Group C, cured; Group NC, non-cured; ESR, erythrocyte sedimentation ratio (normal range <20 mm/h); CRP, C-reactive protein (normal range of <0.5 mg/dL); SUV_max_, maximum standardized ^18^F-fluorodeoxyglucose uptake value on PVO lesion; * *p* < 0.001 between groups C and NC; *p*-values of <0.05 were considered statistically significant.

**Table 4:** Performances of the DNN models for predicting remission in PVO.

DNN Models	Sensitivity	Specificity	PPV	NPV	Accuracy	AUC
DNN model 1 (ESR and CRP)	75%	78.6%	85.7%	64.7%	76.3%	0.768 [0.625–0.910]
DNN model 2 (ESR and SUV_max_)	75%	85.7%	90%	66.7%	79%	0.804 [0.674–0.933]
DNN model 3 (CRP and SUV_max_)	91.7%	78.6%	88%	84.6%	86.8%	0.851 [0.726–0.976]
DNN model 4 (ESR, CRP, and SUV_max_)	87.5%	92.7%	95.5%	81.3%	89.5%	0.902 [0.804–0.999]

DNN, deep neural network; PPV, positive predictive value; NPV, negative predictive value; AUC, area under the receiver operating characteristic; ESR, erythrocyte sedimentation ratio; CRP, C-reactive protein; SUV_max_, maximum standardized ^18^F-fluorodeoxyglucose uptake value on PVO lesion; [ ], 95% confidence intervals.

**Table 5:** Incorrect predictions in DNN model 4.

Number of Case	ESR	CRP	SUV_max_	Prediction of DNN Model 4	Actual Result
# 33	79	5.104	6.38	Non-cured	Cured
# 45	25	0.537	7.79	Non-cured	Cured
# 107	97	3.222	6.2	Non-cured	Cured
# 126	7	0.149	4.6	Cured	Non-cured

DNN, deep neural network; ESR, erythrocyte sedimentation ratio (normal range <20 mm/h); CRP, C-reactive protein (normal range <0.5 mg/L); SUV_max_, maximum standardized ^18^F-fluorodeoxyglucose uptake value on PVO lesion.

## Data Availability

The datasets acquired and analyzed during the current study are available from the corresponding author on reasonable request.
